# Genetic polymorphisms leading to altered T-cell and dendritic cell function cooperate to produce expansion of proinflammatory T-cell subsets in NZB c1 congenic mice

**DOI:** 10.1186/ar3962

**Published:** 2012-09-27

**Authors:** N Talaei, C Landolt-Marticorena, B Noamani, E Pau, N-H Chang, JE Wither

**Affiliations:** 1 Toronto Western Research Institute, University of Toronto, ON, Canada

## Background

We have previously shown that B6 mice with an introgressed homozygous NZB chromosome 1 (c1) interval (70 to 100 cM) develop high titers of antinuclear antibodies and severe glomerulonephritis. Using subcongenic mice with shorter c1 intervals, we found that expansion of T_H_1, T_H_17, and T_FH _cells was closely associated with the severity of glomerulonephritis. The same expansions were observed using ovalbumin (OVA) as an exogenous antigen, indicating that this was an intrinsic aspect of their immune system. Here we have investigated the role of T cells and dendritic cells (DC) in this expansion.

## Methods

OVA-specific T cells from B6 or c1 congenic OT-II TCR transgenic mice were adoptively transferred into B6.Thy1.1 or c1(70-100).Thy1.1 mice. The mice were immunized with OVA emulsified in CFA, sacrificed 2 weeks later, and the proportion of various splenic T-cell subsets determined by flow cytometry, gating on Thy1.2^+ ^(transferred) T cells. Bone marrow-derived DC isolated from 8-week-old c1(70-100), c1(88-100) and c1(96-100) congenic and B6 control mice were cultured in the presence of LPS, imiquimod and CpG, or pulsed with OVA and co-cultured with naïve OT-II T cells. Production of cyto/chemokines (IL-12, IL-23, IL-6) by stimulated DC was analyzed by ELISA or flow cytometry.

## Results

Adoptive transfer experiments revealed that the increased IFNγ and IL-17 secreting cell differentiation in c1(70-100) congenic mice arises in part from intrinsic T-cell defects localizing to the NZB c1 96 to 100 and 88 to 96 intervals, respectively. However, OT-II T cells from all mouse strains examined demonstrated enhanced differentiation to T_H_1, T_H_17, and T_FH _populations when transferred into c1(70-100).Thy1.1 as compared with B6.Thy1.1 mice. Since DC play an important role in the antigen presentation and cytokine secretion that directs T-cell responses, DC function was contrasted in the various mouse strains. Following TLR stimulation, DC from c1(70-100) mice expressed significantly higher levels of MHC and co-stimulatory molecules, and secreted higher amounts of proinflammatory cytokines such as IL-6 and IL-12. Consistent with altered DC function, OVA pulsed DC from c1(70-100) mice induced significantly increased differentiation of naïve OT-II cells to IFNγ, IL-17 or IL-21 secreting cells as compared with B6 DC.

## Conclusion

Our results suggest that a genetic polymorphism in the 70 to 100 interval of NZB c1 congenic mice alters DC function and acts together with intrinsic T-cell defects that map to the 88 to 100 interval to promote the expansion of T_H_1, T_H_17 and T_FH _cells in c1(70-100) mice.

